# Next-Generation Sequencing in Lung Cancers—A Single-Center Experience in Taiwan

**DOI:** 10.3390/medicina60020236

**Published:** 2024-01-29

**Authors:** Wei-An Lai, Yen-Shuo Huang, Kung-Chao Chang, Sheau-Fang Yang, Chih-Jen Yang, Yu-Wei Liu, Huan-Da Chen

**Affiliations:** 1 Department of Pathology, Kaohsiung Medical University Hospital, Kaohsiung Medical University, Kaohsiung 80708, Taiwan; eddie83a@gmail.com (Y.-S.H.);; 2 Department of Pathology, National Cheng Kung University Hospital, College of Medicine, National Cheng Kung University, Tainan 70101, Taiwan; 3 Department of Pathology, Kaohsiung Medical University Hospital, College of Medicine, Kaohsiung Medical University, Kaohsiung 80708, Taiwan; 4 Center for Cancer Research, Kaohsiung Medical University, Kaohsiung 80708, Taiwan; 5 Division of Pulmonary and Critical Care Medicine, Department of Internal Medicine, Kaohsiung Medical University Hospital, Kaohsiung Medical University, Kaohsiung 80708, Taiwan; 6 School of Post-Baccalaureate Medicine, College of Medicine, Kaohsiung Medical University, Kaohsiung 80708, Taiwan; 7 Division of Thoracic Surgery, Department of Surgery, Kaohsiung Medical University Hospital, Kaohsiung Medical University, Kaohsiung 80708, Taiwan

**Keywords:** lung cancer, next-generation sequencing, co-occurring mutation

## Abstract

*Background and Objectives*: Lung cancer is a leading cause of cancer mortality in Taiwan. With rapid advancement of targeted therapeutics in non-small cell lung cancers, next-generation sequencing (NGS) is becoming an important tool for biomarker testing. In this study, we describe institutional experience of NGS analysis in non-small cell carcinoma (NSCLC). *Materials and Methods*: A cohort of 73 cases was identified from the institutional pathology archive in the period between November 2020 and December 2022. *Results*: Adenocarcinoma was the most common histologic type (91.8%). Most patients presented with stage IIIB and beyond (87.7%). Twenty-nine patients (39.7%) were evaluated at the time of initial diagnosis, while the others had received prior chemotherapy or targeted therapy. The most frequently mutated gene was EGFR (63%), and this was followed by TP53 (50.7%), KRAS (13.7%), RB1 (13.7%), and CDKN2A (13.7%). Clinically actionable mutations associated with a guideline-suggested targeted therapy were identified in 55 cases (75.3%) overall, and in 47.1% of cases excluding EGFR TKI-sensitizing mutation. Biomarkers other than EGFR TKI-sensitizing mutations were compared. Cases without TKI-sensitizing EGFR mutation had more level 1 or 2 biomarkers (excluding EGFR TKI-sensitizing mutations) than cases with TKI-sensitizing EGFR mutations (47.1% versus 20.1%, *p* = 0.016). Progressive disease was associated with co-occurrence of clinically actionable mutations (20.5% versus 0%, *p* < 0.05). Eight of the nine cases with co-occurring actionable genetic alternations had an EGFR mutation. After an NGS test, 46.1% of actionable or potentially actionable genetic alternations led to patients receiving a matched therapy. *Conclusions*: Our study demonstrated that NGS analysis identifies therapeutic targets and may guide treatment strategies in NSCLC. NGS tests may be advantageous over multiple single-gene tests for optimization of treatment plans, especially for those with non-EGFR mutations or those with progressive disease.

## 1. Introduction

Cancers are the leading cause of death in Taiwan. The increase in lung cancer incidence and mortality in Taiwan is in line with the global trend. The age-adjusted incidence leaped from 16.5 to 37 cases per 100,000 from 1986 to 2017 [1]. Among the 10 most common cancers, lung cancer has the lowest 5-year survival rate. In Taiwan, more than half of lung cancer patients are never-smokers and being female is a significant risk factor [1]. Most patients (70%) present with unresectable disease at stage III or IV [1]. For this patient population, biomarker testing has become standard to guide treatment. Identification of molecular targets including EGFR, ALK, and ROS1 has been traditionally performed using single-gene test methods such as PCR and fluorescence in situ hybridization (FISH) analysis. In the Taiwanese population, EGFR tyrosine kinase inhibitor (TKI)-sensitizing mutation rates ranged 55~56% [1,2,3]. The *EGFR* mutation rate of the Taiwanese population is in line with reports of the East Asian populations and higher than that of the Caucasian population (10~20% mutation rate) [4]. *ALK* mutation was identified in 10% of the *EGFR* wild-type lung adenocarcinoma in Taiwan. Other driver mutations reported include *KRAS* (7%), *HER2* (6%), *BRAF* (4%), *ROS1* (3%), and *MET* (2%) [5].

Screening of these mutations is implemented by the National Health Insurance program in Taiwan. As therapeutics evolve, more targeted therapies for other less common mutations in advanced lung cancer are becoming available and recommended by clinical guidelines such as the guidelines for the National Comprehensive Cancer Network (NCCN), and the European Society for Medical Oncology (ESMO). These mutations include *RET* rearrangement, *NTRK* rearrangement, *BRAF* V600E mutation, *KRAS* G12C mutation, *MET* exon 14 skipping mutations, *MET* amplifications, *EGFR* exon 20 insertions, and *HER2* mutations [6,7]. Empowering the capability of molecular diagnosis with an appropriate range of detection is thus crucial for the selection of the best therapeutic option for cancer treatment. In this scenario, next-generation sequencing (NGS) may be advantageous over single-gene tests. A cancer-related panel allows detection of an array of relevant target genes in a single test. It thus reduces turnaround time and the amount of tissue required as compared to multiple single-gene assays.

Despite the advantages of NGS in implementing biomarker identification in lung cancer, its use is limited because of the relatively high cost and exclusion of the National Health Insurance reimbursement in Taiwan. In this study, we retrospectively searched the institutional pathology archive for all NGS reports of non-small cell lung cancers (NSCLC). We describe the clinicopathological attributes and mutational profiles and hope to reflect the real-world experience of the utility of NGS in NSCLC. In this study, we demonstrate the usefulness of NGS in identification of clinically actionable targets, as defined and categorized by the OncoKB Precision Oncology Knowledge Base [8]. We also report association of co-occurring driver mutations in recurrent diseases.

## 2. Materials and Methods

### 2.1. Data Collection

This is a retrospective study based on NGS reports retrieved from the institutional pathology archive in the period between November 2020 and December 2022. This study was conducted in accordance with the Declaration of Helsinki and approved by the Institutional Review Board (IRB No. KMUHIRB-E(I)-20230095). In Taiwan, analysis of EGFR, ALK, and ROS1 mutational status was implemented by a National Health Insurance program. NGS tests are self-reimbursed and suggested in clinical scenarios when EGFR, ALK, and ROS1 mutations are not identified, or when patients experience progressive disease with treatment failure. A total of 73 NSCLCs analyzed using NGS were identified. All cases had a corresponding histopathology report, with diagnoses of NSCLC made by institutional pathologists according to the 2021 WHO Classification of thoracic tumors [9]. The formalin-fixed paraffin embedded (FFPE) specimen adequacy was determined by an institutional pathologist, and the NGS analysis was performed in a College of American Pathologist (CAP)-accredited NGS laboratory at ACT Genomics (ACT Genomics Co., Taipei, Taiwan). Single-nucleotide variants (SNV), small insertions and deletions (indels) and copy-number variations (CNV) were detected using the ACTOnco or ACTDrug panels. The ACTOnco is a comprehensive cancer gene panel encompassing the coding region of 440 cancer-related genes. The ACTDrug is a targeted panel encompassing 40 potentially actionable genes. The ACTFusion panel, an RNA-based NGS detecting 13 targetable fusions was implemented for all FFPE specimens that had passed quality control for RNA content. These tests provide uniform coverage of the targeted regions, enabling target base coverage at 100 x ≥ 85% with a mean coverage ≥ 500 x. 

Detected variants with clinical significance were reported and signed off by institutional pathologists. A retrospective review of these reports was performed. Data including histopathologic diagnosis, detected gene variants, gender, age, tumor site, and specimen type were collected. Clinical records were reviewed for cancer stage and whether the patient had received a matched targeted therapy.

### 2.2. Stratification of Genetic Alternations

The potentially actionable genetic alternations were stratified into four categories by therapeutic levels of evidence based on published clinical evidence and guidelines. The search for such information was performed through the OncoKB Precision Oncology Knowledge Base (https://oncokb.org, accessed on 6 October 2023) [8]. Level 1 genetic alternations include FDA-approved mutations and fusions of copy-number alternations that predict the response to an FDA-approved therapeutic treatment. Level 2 alternations include those that are standard-of-care biomarkers that predict response to an FDA-approved therapeutic recommended by NCCN or other clinical treatment guidelines for NSCLC. Level 3 genetic alternations encompass those with clinical evidence that the biomarker is associated with a drug response in patients but has not been included in the standard of care. Level 4 alternations are investigational markers associated with a biological response. The stratification is in accordance with OncoKB™ Therapeutic Level of Evidence V2, accessed on 6 October 2023 (https://www.oncokb.org/therapeutic-levels, accessed on 6 October 2023).

### 2.3. Statistical Analyses

Categorical data were analyzed via Chi-squared test. The *p*-values were two-sided, and a value < 0.05 was considered significant. The analysis was performed using R software (version 3.5.1, R Foundation for Statistical Computing, Vienna, Austria).

## 3. Results

### 3.1. Clinicopathological Profile

A total of 73 cases were included in the cohort (Table 1). The patients’ ages ranged from 42 to 86 years (median 66). The female to male ratio was 1.15 to 1. Adenocarcinomas were the most frequent diagnosis (67 cases, 91.8%) per 2021 WHO classification [9]. Other histopathological distributions were listed in Table 1. Most patients initially presented with advanced unresectable disease (≥stage IIIB, 87.7%). Twenty-nine cases (39.7%) were evaluated at the time of initial diagnosis, while the other forty-four cases (60.3%) were evaluated as the disease progressed, having received prior chemotherapy or targeted therapy. The pathologic specimens were obtained from primary sites in 50 cases (68.5%) and from metastatic sites in 23 cases (31.5%). They comprised 40 biopsies (54.8%), 29 resections (39.7%), and 4 cell blocks (5.5%).

### 3.2. Overall Mutational Profile

Among the cohort, mutations were identified in 75 genes (Table S1). The most frequently mutated gene was *EGFR* (63%) and this was followed by *TP53* (50.7%), *KRAS* (13.7%), *RB1* (13.7%), and *CDKN2A* (13.7%) (Table 2). Among the 73 cases, 68 cases had one or more mutations identified.

**Figure 1:**
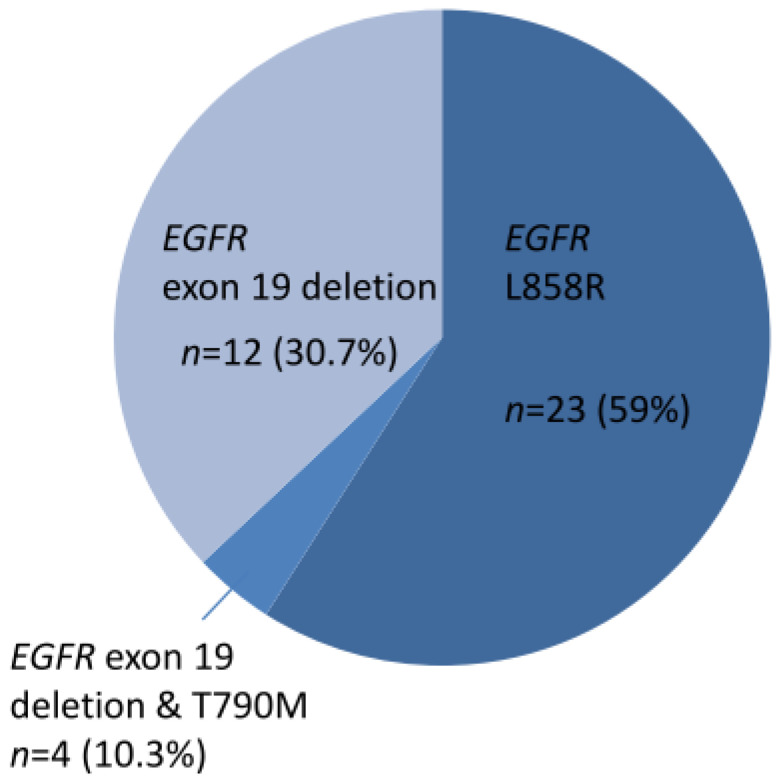
Thirty-nine cases of NSCLC with EGFR TKI-sensitizing mutations.

**Figure 2:**
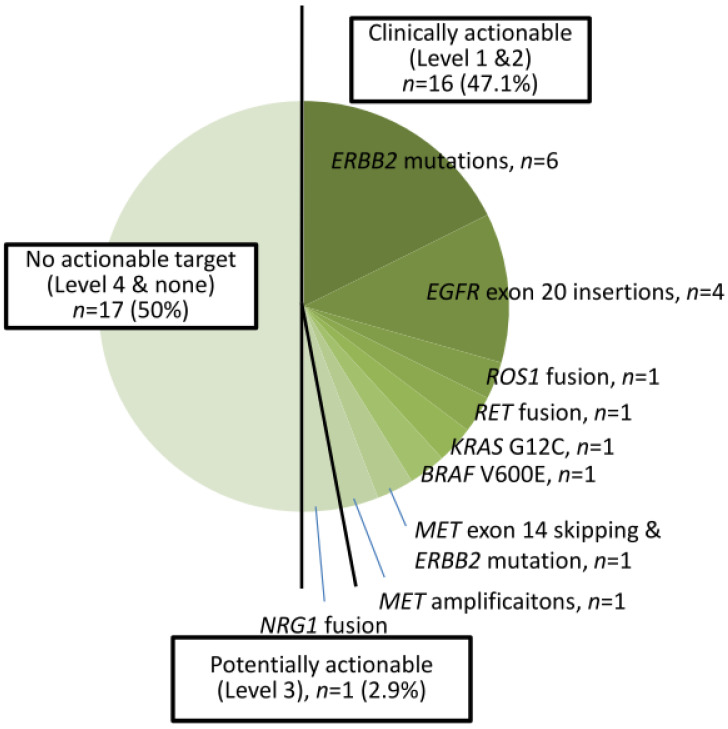
Thirty-four cases of NSCLC with non-EGFR TKI-sensitizing mutations.

A total of 39 cases harbored *EGFR* TKI-sensitizing mutations, including 23 *EGFR L858R* and 16 exon 19 deletions (Figure 1). TKI-resistant *EGFR* T790M mutations were present in four cases. Among these 39 cases, 9 harbored co-occurring level 1 and level 2 targetable mutations defined by the OncoKB database. These included five *MET* amplifications, two *ERBB2* amplifiations, and one *BRAF* V600E mutation (supplementary Figure S1). Of the 34 cases without TKI-sensitizing *EGFR* mutations, OncoKB-defined level 1 and level 2 oncogenic drivers associated with clinically actionable therapies were identified in 16 cases (47.1%) (Figure 2 and Figure S2). These included six *ERBB2* mutations, four *EGFR* exon 20 insertions, one *ROS1* fusion, one *RET* fusion, one *BRAF* V600E, one *KRAS* G12C, and one *MET* exon 14 skipping mutation plus *ERBB2* mutation, and one *MET* amplification (Figure 2). 

Overall, 55 cases (75.3%) harbored level 1 or 2 biomarkers associated with a clinical guideline-recommended targeted therapy. One case (1.4%) harbored *NRG1* fusion, a level 3 biomarker with potential actionable therapeutics. Seventeen cases (23.3%) were found to have no clinically actionable events (level 4 or none).

### 3.3. Subgroup Analysis of Biomarker Frequency

The primary use of NGS is to identify non-*EGFR* TKI-sensitizing therapeutic targets. Thus, biomarkers other than *EGFR* TKI-sensitizing mutations were compared between groups with or without *EGFR* mutations. Cases without TKI-sensitizing *EGFR* mutations had more level 1 or 2 biomarkers (excluding TKI-sensitizing *EGFR* mutations) than cases with TKI-sensitizing *EGFR* mutations (47.1% versus 20.1%, *p* = 0.016, Table 3). The frequencies of level 3 and level 4 biomarkers were not significantly different between the two groups. 

For progressive diseases, identification of additional therapeutic targets is key for further treatment options. Therefore, we explored the presence of co-occurring biomarkers. Compared to primary disease, progressive disease was associated with co-occurring level 1 or 2 biomarkers (20.5% versus 0%, *p* = 0.0093, Table 4). And among the nine cases with co-occurring mutations, eight harbored both an *EGFR* TKI-sensitizing mutation and another driver such as *BRAF* V600E mutation (1/8), *MET* amplification (5/8), or *ERBB2* amplification (2/8). There was no case presenting with co-occurrence of a level 3 biomarker with a level 1 or 2 biomarker. There were no co-occurring level 3 biomarkers. The co-occurrent rates of a level 4 biomarker with a level 1 or 2 biomarker were not significantly different in patients with primary or progressive diseases (13.8% versus 13.6%, *p* = 0.9848).

### 3.4. Use of Matched Therapy

**Figure 3:**
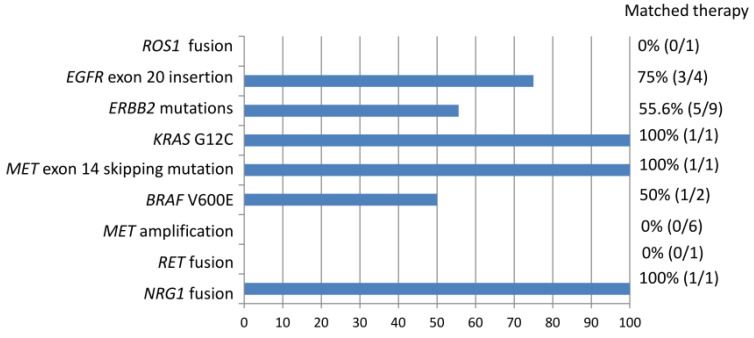
Non-*EGFR* TKI-sensitizing mutations identified in the entire cohort associated with matched therapy.

Among the entire cohort, 1 level 1 mutation (excluding *EGFR* TKI-sensitizing mutation) 24 level 2 mutations, and 1 level 3 mutations were identified (Figure 3). Based on the NGS results, 46.1% of actionable (level 1 and 2) and potentially actionable (level 3) genetic alternations led to the patient receiving a matched therapy. Overall, 12 of the 73 patients (16.4%) received a matched therapy. Among patients with *EGFR* exon 20 insertions and *ERBB2* mutations, 75% and 55.6% received targeted therapeutics including afatinib, trastuzumab deruxtecan, and mobocertinib (TAK-788), respectively. One of the two patients with *BRAF* V600E mutations was treated with dabrafenib and trametinib. Tumors with *KRAS* G12C, *MET* exon 14 skipping mutation, or *NRG1* fusion were each treated by targeting the agents sotorasib, capatinib, and afatinib. *ROS1* fusion was detected in one case that had not been identified via immunohistochemistry. However, the patient died before the NGS results were obtained. The one patient with *RET* fusion was referred to another hospital for further treatment and was lost to follow up in our hospital. Detection of *MET* amplification did not lead to the use of *MET*-targeting agents.

## 4. Discussion

With the rapid evolution of target therapy in NSCLC and advances in comprehensive genomic profiling, we are transitioning to an era of personalized medicine. While mutational analysis for *EGFR*, *ALK*, and *ROS1* are standard for unresectable NSCLC [10,11], NGS analysis has not become routine and is not reimbursed by the National Health Insurance Program in Taiwan. In clinical scenarios, NGS analyses are performed in selective cases, mostly those without *EGFR* TKI-sensitizing mutations, *ALK* gene fusions, or *ROS1* gene fusions, as well as those with treatment failure and experiencing progressive disease. In this study, we describe a cohort of institutional NSCLC patients who had FFPE specimens submitted for NGS tests through the institutional pathology department in a clinical setting. Between the study periods of November 2020 and December 2022, a total of 112 patients were diagnosed with advanced NSCLC requiring molecular tests for further treatment. Twenty-nine cases have undergone next-generation sequencing within this period. 

The 72 cases identified in our cohort had a similar genomic background as compared to previous studies, as we identified the most commonly mutated genes to be *EGFR*, *TP53*, *KRAS, RB1*, and *CDKN2A* [12,13,14]. Our cohort had similar, albeit slightly higher, mutation rates of *EGFR* (63%), *TP53* (50.7%), and *KRAS* mutation (13.7%) as compared to a study involving subjects of East Asian ethnicity (*EGFR* 51.1%, *TP53* 49.1%, *KRAS* 9.3%) [4]. The similarity of genetic mutation rates suggests that, although the cases in our cohort are not random, the genetic background did not deviate much from the molecular epidemiology of NSCLC. Importantly, our study demonstrates that overall, actionable level 1 and 2 clinically targetable biomarkers, including *EGFR* TKI-sensitizing mutations, *EGFR* exon 20 insertions, *ROS1* fusion, *RET1* fusion, *KRAS G12C*, *BRAF V600E*, and *MET* exon 14 skipping mutations, *MET* amplifications, and *ERBB2* mutations were identified in 75.3% of cases. Our study suggests that, with a single NGS test, these patients may benefit from a guideline-suggested treatment [15,16]. 

Notably, we demonstrated the utility of NGS in identifying OncoKB database-defined level 1 and level 2 biomarkers other than *EGFR* TKI-sensitizing mutations. In non-EGFR-mutated cases, a clinically actionable therapeutic target could be detected in up to 47.1% cases. These include a level 1 biomarker *ROS1* fusion. In our institution, non-*EGFR*-mutated cases are screened via immunohistochemistry for ROS1 overexpression. Those with ROS1 expression over the positive threshold are subsequently tested via in situ hybridization for *ROS1* fusion. A lower-than-threshold immunohistochemical staining result of ROS1 was reported in this case, which was considered a false negative when the NGS result was obtained. This example demonstrated the value of an NGS test that may identify genetic alternations with superior sensitivity as compared to traditional methods, leading to identification of appropriate treating targets. Sixteen level 2 biomarkers that predict response to an FDA-approved therapeutic recommended by clinical treatment guidelines were identified in non-*EGFR*-mutated cases. These included seven *ERBB2* mutations, four *EGFR* exon 20 insertions, one *RET1* fusion, one *KRAS G12C*, one *BRAF V600E*, one *MET* exon 14 skipping mutation, and one *MET* amplification. While *RET* fusion, *BRAF V600E*, and *MET* exon 14 skipping mutations are recommended by the NCCN as first-line therapies for advanced NSCLC, the others may serve as subsequent-line therapies after systemic treatment [15]. Moreover, a level 3 marker, *NRG1* fusion, was identified in this cohort. *NRG1* fusion generates the retained extracellular EGF-like domain of NRG1 and transmembrane component of the specific fusion partner [17]. The *NRG1* fusion partner is diverse, with the most frequent partner being *CD71*. The *NRG1-SLC3A2* fusion detected in our cohort had been reported in NSCLC previously [17,18,19]. NRG1 fusion proteins serve as ligands for ERBB3 and ERBB4. Upon NRG1 ligand binding, ERBB3 heterodimerizes with ERBB2, and the ERBB2-ERBB3 complex upregulates the downstream PI3K/Akt pathway [17,18,20]. Afatinib is a second-generation *EGFR* TKI, which is an irreversible inhibitor that targets wild-type *EGFR* and mutant *EGFR*, *ERBB2,* and *ERBB4*, thus blocking all possible homodimers and heterodimers of the ERBB family receptor [21]. Case reports have demonstrated the therapeutic roles of afatinib and other ERBB-targeting agents in NSCLC with *NRG1* fusion [19,22,23,24]. Afatinib is the most frequently used therapeutic in these reports. Partial responses were achieved for up to 12 months in lung adenocarcinoma and 10 months in invasive mucinous adenocarcinoma of the lung harboring *NRG1* fusion. Patients with advanced NSCLC positive for *NRG1* fusion are currently being investigated in the TAPUR clinical trial (Group 18: *NRG1*) (NCT02693535) for afatinib treatment. 

In this study, we also demonstrated that progressive diseases had more co-occurring level 1 and level 2 mutations as compared to primary disease (20.5% vs. 0%, *p* = 0.0093). This indicates that as compared to primary disease, targetable mutations could be diverse and an NGS test in this scenario may expand treatment strategies and help optimize the choice of therapeutic agent. Also, increased co-occurring mutations in progressive disease may reflect selection pressure on tumor cells with systemic treatments resulting in tumor heterogeneity. And among the nine cases with co-occurring level 1 and level 2 mutations, eight harbored an *EGFR* TKI-sensitizing mutation. The eight co-occurring mutations associated with *EGFR* TKI-sensitizing mutations include five *MET* amplifications, two *ERBB2* amplifications, and one *BRAF* V600E mutation. This is in line with a previous study showing that in NSCLC patients with co-occurring actionable drivers, 80% harbored an *EGFR* mutation. It was found that concurrent *MET* or *ERBB2* mutations contribute to the resistance to TKI in *EGFR*-mutated patients [25,26,27]. *ERBB2* mutations and *MET* amplification, which may act through ERBB3, drive activation of PI3K/AKT signaling and thus bypass EGFR signaling, conferring EGFR TKI resistance [28]. While ERBB2-targeting agents, including trastuzumab, deruxtecan, and ado-trastuzumab emtansine, are FDA-approved agents for the treatment of NSCLC patients who had received prior systemic therapy [29,30], MET amplification is currently included in the NCCN guideline as an emerging marker with available targeting agents including capmatinib, tepotinib, and crizotinib [31,32].

In our cohort, NGS analysis identified 1 level 1 mutation (*ROS1* fusion), 24 level 2 mutations (*ERBB2* mutations, *EGFR* exon 20 mutation, *RET* fusion, *KRAS* G12C, *BRAF* V600E, *MET* exon 20 skipping mutation, *MET* amplification), and 1 level 3 mutation (*NRG1* fusion) (Figure 3). According to the NGS results, 12 genetic alternations (46.1%) received a matched targeted therapy. *ROS1* fusion is an FDA-approved biomarker predictive of drug response. The case with *ROS1* fusion did not receive a matched therapy because the NGS result came out too late and the patient died of lung cancer. Level 2 biomarkers are defined as standard-of-care biomarkers recommended by the NCCN guideline or other professional guidelines predictive of response to an FDA-approved drug. In our cohort, only a subset of patients with a level 2 biomarker received a matched therapy. This may be attributed to relatively recent FDA approval of the targeting agents in this category and limited access to these agents outside the context of clinical trials. For example, targeted treatments for exon 20 insertions of *EGFR* and *ERBB2* have only recently been approved by the FDA for the treatment of NSCLC carrying these mutations. Mobocertinib is an irreversible tyrosine kinase inhibitor which targets EGFR and ERBB2 receptors. It binds the exon 20 insertions at lower concentrations than wild-type receptors. Reports from the Study AP32788-15-101 led to FDA approval of mobocertinib for locally advanced or metastatic NSCLC with EGFR exon 20 insertion on September 15, 2021 [33]. Trastuzumab deruxtecan is an antibody–drug conjugate composed of a humanized monoclonal antibody against HER2 (trastuzumab) complexed with a topoisomerase I inhibitor (deruxtecan) [34]. The success of its use in HER2-mutant NSCLC was demonstrated in the DESTINY-Lung02 phase II study, leading to FDA-accelerated approval of its use for previously treated metastatic or unresectable NSCLC on 11 August 2022 [35]. It is the first FDA-approved agent for *HER2*-mutant NSCLC. Access to these targeted therapies is limited outside of clinical trial contexts before these dates. For another example, the patient with *RET* fusion was not treated in our institution as no targeting agent has been approved by the Taiwan FDA at that time. The patient was referred to another medical center to participate in a clinical trial for the use of appropriate target therapy. Finally, the NCCN guideline listed *MET* amplification as an emerging biomarker to identify novel therapies for NSCLC [15]. The effectiveness of MET-selective TKI has been evaluated in several studies. Patients with high-level *MET* amplification are associated with clinical benefits as opposed to those with low-level amplification in response to MET inhibitors including campmatinib, tepotinib, and crizotinib [36,37,38]. However, there is no standardized method for determining *MET* amplification. The definition of “high-level” amplification is evolving and the threshold may be different depending on the assaying method [31]. Although MET-targeting therapy has emerged, more investigation is warranted for standardization of biomarker detection and evaluation of treatment response. Cases with *MET* amplification detected via NGS may benefit from MET-targeting therapy in the near future. 

There were drawbacks of this study. The sample size is small and as NGS is not nationally reimbursed, the inclusion of cases may not be random. However, as discussed above, the genetic epidemiology of the current cohort did not deviate much from that of the East Asian ethnicity. Integration of multi-institutional data in Taiwan or regionally in East Asia is to be pursued in the future. This study reflects real-world observation with data generated from patients with NSCLC requiring an NGS for further clinical management from a single medical center in Taiwan. This study demonstrated that a clinically actionable and potentially actionable mutation may be identified in 75.3% of patients with NSCLC. Excluding cases with *EGFR* TKI-sensitizing mutations, actionable mutations may be identified in up to 47.1% of cases. Furthermore, co-occurring actionable mutations are identified in 20.5% of cases, and most are associated with an *EGFR* TKI-sensitizing mutation. A matched therapy is used in 46.1% of actionable or potentially actionable genetic alternations according to the NGS test results. The percentage is expected to increase as more and more emerging targeting therapeutics are being evaluated, such as those for *MET* amplification. An NGS test is advantageous for the identification of one or more targetable mutations as well as potential resistance mechanisms and this may facilitate the optimization of clinical treatment decisions.

## 5. Conclusions

With the variety and prevalence of targetable mutations in NSCLC, NGS is advantageous over multiple single tests for comprehensive genomic profiling. About half of the identified actionable or potentially actionable genetic alternations led to patients receiving a matched therapy after an NGS test, and the proportion is expected to be higher in the future. NSCLC cases with non-*EGFR* TKI-sensitizing mutations or those with progressive disease may benefit particularly from an NGS test. 

## Figures and Tables

**Table 1:** Clinicopathological feature of NSCLC cohort.

		Case No., (%)
Age	42–86 (median 66)	
Gender		
	Female	39 (53.4)
	Male	34 (46.6)
Histopathology		
	Adenocarcinomas	67 (91.8)
	Squamous cell carcinoma	2 (2.7)
	Adenosquamous carcinoma	1 (1.4)
	Lymphoepithelial carcinoma	1 (1.4)
	Large cell neuroendocrine carcinoma	1 (1.4)
	Sarcomatoid carcinoma	1 (1.4)
Stage		
	≤IIIA	9 (12.3)
	≥IIB	64 (87.7)
Disease status at specimen acquisition		
	Initial diagnosis	29 (39.7)
	Progressive disease	44 (60.3)
Specimen site		
	Lung	50 (68.5)
	Metastatic sites	23 (31.5)
Specimen type		
	Biopsy	40 (54.8)
	Resection	29 (39.7)
	Cell block	4 (5.5)
NGS panel		
	ACTDrug (targeted)	56 (76.7)
	ACTOnco (comprehensive)	17 (23.3)

**Table 2:** Frequencies of mutated genes in NSCLC cohort.

	Case No.	%
*EGFR*	46	63
*TP53*	37	50.7
*KRAS*	10	13.7
*RB1*	10	13.7
*CDKN2A*	10	13.7
*ERBB2*	8	11
*PIK3CA*	8	11
*MET*	7	9.6
*CDK4*	6	8.2

**Table 3:** Comparison of potentially actionable mutations between TKI-sensitizing *EGFR*-mutated cases and non-mutated cases.

	*EGFR*	Non-*EGFR*	
	*n* (%)	*n* (%)	*p* Value
Level 1 and 2			0.016
Yes	8 (20.1)	16 (47.1)	
No	31 (79.9)	18 (52.9)	
Level 3 only			
Yes	0 (0)	1 (2.9)	0.2425
No	39 (100)	33 (97.1)	
Level 4 only			0.9937
Yes	8 (20.1)	7 (20.1)	
No	31 (79.9)	27 (79.9)	

**Table 4:** Comparison of potentially actionable mutations between initial diagnosis and progressive disease cases.

	Initial Diagnosis *n* (%)	Progressive Disease *n* (%)	*p* Value
Level 1 and 2			0.0093
Yes	0 (0)	9 (20.5)	
No	29 (100)	35 (79.5)	
Level 3 only			NA
Yes	0 (0)	0 (0)	
No	29 (100)	44 (100)	
Level 4 only			0.9848
Yes	4 (13.8)	6 (13.6)	
No	25 (86.2)	38 (86.4)	

## Data Availability

The data presented in this study are available in Table 1, Figure 1 and Figure 2, and Table S1.

## Supplementary Materials

The following supporting information can be downloaded at: https://www.mdpi.com/article/10.3390/medicina60020236/s1. Figure S1: Genetic landscape of NSCLC with *EGFR* TKI mutations; Figure S2: Genetic landscape of NSCLC without EGFR TKI mutations; Table S1: Frequencies of mutated genes detected by NGS.
