# Supplementary material for: Next-Generation Sequencing in Lung Cancers—A Single-Center Experience in Taiwan
Source: Medicina (Kaunas). 2024 Jan 29;60(2):236. doi: 10.3390/medicina60020236 (PMC10890140; doi:10.3390/medicina60020236)
Supplement: Supplementary file 1 [file medicina-60-00236-s001.zip › Supplementary figures.pdf]

Supplementary figure S1

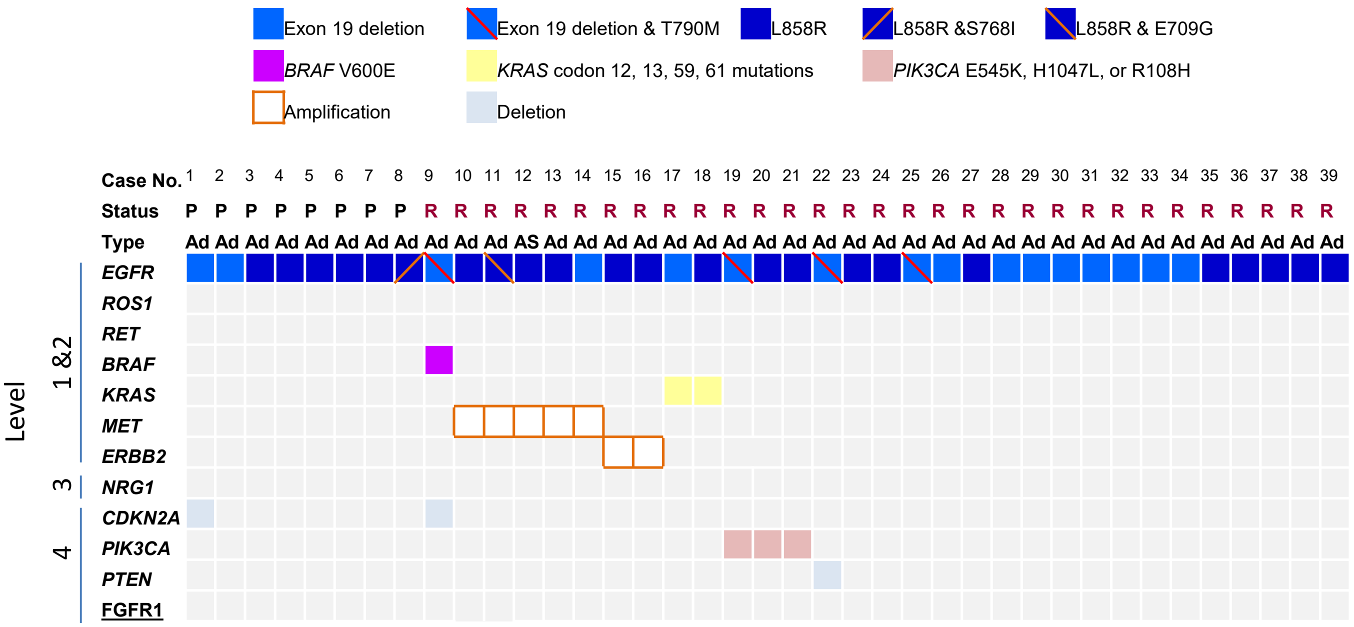

Supplementery figure S1. Genetic landscape of NSCLC with *EGFR* TKI mutations. R, progressive disease; P, primary disease; AS, adenosquamous carcinoma; Ad, adenocarcinoma.

Supplementary figure S2

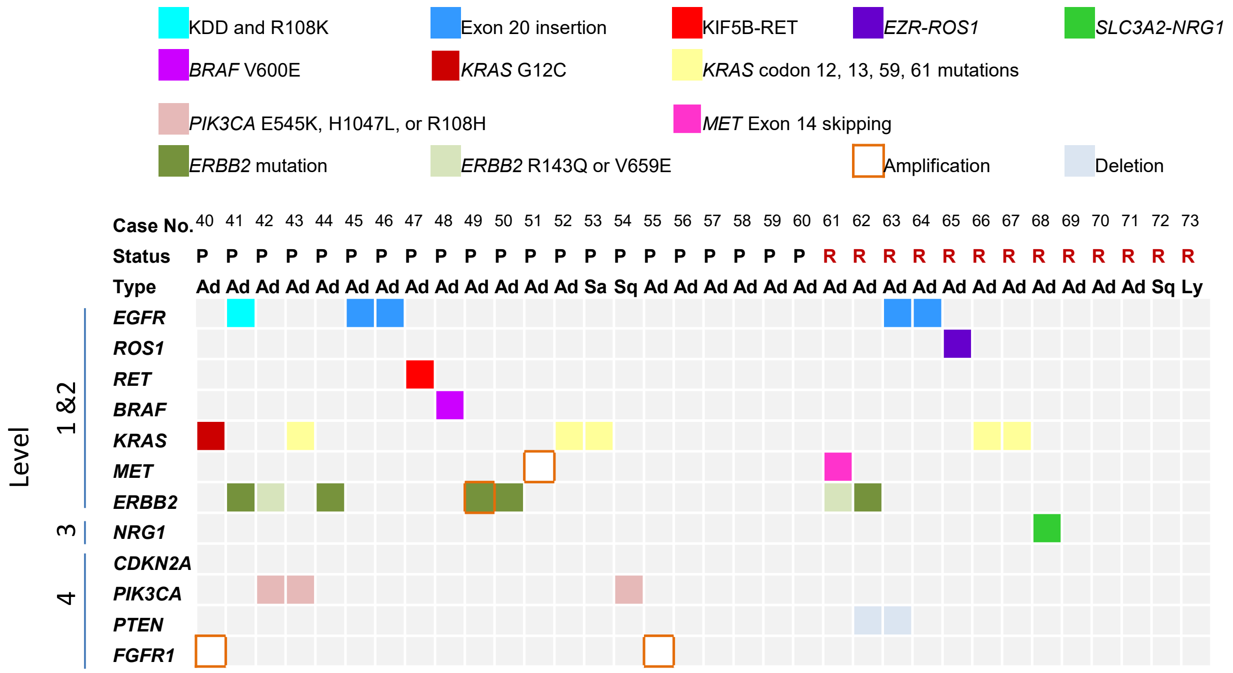

Supplementery figure S2. Genetic landscape of NSCLC without *EGFR* TKI mutations. R, progressive disease; P, primary disease; Ad, adenocarcinoma; Sa: sarcomatoid carcinoma; Sq, squamous cell carcinoma; Ly, lymphoepithelial carcinoma.
